# Prenatal NSAIDs exposure and childhood kidney disease: a systematic review and meta-analysis

**DOI:** 10.3389/fped.2026.1808919

**Published:** 2026-05-21

**Authors:** Xinping Cui, Rong Feng, Bin Song, Qian Zhou, Yan Zeng

**Affiliations:** 1 North Sichuan Medical College, Nanchong, China; 2 People’s Hospital of Deyang City, Deyang, China; 3 Southwest Medical University, Luzhou, China; 4 Department of Pediatrics, People’s Hospital of Deyang City, Deyang, China

**Keywords:** children, kidney disease, meta-analysis, NSAIDs, risk factors

## Abstract

**Objective:**

Studies have shown a link between prenatal NSAIDs exposure and childhood kidney disease; however, consensus is lacking. Therefore, we conducted a meta-analysis to assess maternal prenatal exposure to NSAIDs and its relationship with the kidney disease risk in children.

**Methods:**

A systematic search of PubMed, Cochrane Library, Embase, and Web of Science was performed, supplemented by a manual search of references, to identify relevant observational studies for our analysis. Data extraction and quality assessment were independently conducted by two investigators. A random effects meta-analysis was conducted to estimate the summary odds ratio (ORs) and the corresponding 95% confidence interval (CIs).

**Results:**

Seven studies including 4,159,617 participants were selected. The analysis indicated that there was an association between prenatal NSAIDs exposure and kidney disease risk in children (OR 1.36 [1.14–1.62]). After adjusted (OR 2.40 [1.84–3.13]) and unadjusted (OR 1.10 [1.05–1.15]) analyses, the use of NSAIDs during pregnancy was associated with the kidney disease risk in children. Use of NSAIDs in the second and third trimesters of pregnancy is associated with the kidney disease risk in children (second-trimester OR 1.17 [1.09–1.26] and third-trimester OR 1.10 [1.09–1.11]). Sensitivity analysis supported these findings.

**Conclusion:**

With our meta-analysis, we provide evidence for an association between prenatal NSAIDs exposure and kidney diseases in children but do not solve the causality issues concerning potential confounding by other risk factors. More high-quality studies are needed to establish whether the association with NSAIDs is causal.

## 1 Introduction

Kidney diseases in children are a known driver of various health complications and even death, culminating in a substantial socioeconomic burden (1). Currently, the global prevalence of kidney disease in children is steadily increasing (2). The global prevalence of acute kidney injury (AKI) in children is 0.07%, whereas the prevalence of chronic kidney disease (CKD) ranges from approximately 0.3%–1% (2, 3). Genetic and environmental factors may play an important role in kidney disease (4, 5). Studies have demonstrated that exposure to an adverse environment during foetal development is associated with the risk of kidney disease later in life (6). Nonsteroidal anti-inflammatory drugs (NSAIDs), including aspirin, ibuprofen, naproxen, indomethacin, diclofenac, piroxicam, and celecoxib, are among the most commonly prescribed over-the-counter drugs for pregnant women (7, 8). Symptoms that may occur during pregnancy, such as headaches and fevers, can result in NSAIDs exposure (9). Indeed, studies have reported that more than 50% of pregnant women use NSAIDs (10, 11).

Recent studies have shown that there may be a link between NSAIDs use of prenatal and the kidney disease risk in children (10–15). However, other studies have reported inconsistent results (16, 17). In light of the widespread use of NSAIDs, we conducted a meta-analysis to assess maternal exposure to NSAIDs of prenatal and its relationship with the kidney disease risk in children.

## 2 Materials and methods

### 2.1 Research methods

#### 2.1.1 Registration

This meta-analysis was registered on PROSPERO, an international systematic review registry (CRD420251146473). This meta-analysis followed the principles outlined in the Meta-analysis of Observational Epidemiological Studies (MOOSE) guideline (18).

#### 2.1.2 Literature search strategy

We systematically searched the PubMed, Embase, Cochrane Library and Web of Science databases. The literature search covered the period from database inception to 20 April 2026. Search terms encompassed both medical subject headings (MeSH) and free-text terms. These terms were related to NSAIDs and kidney disease and were combined according to the principles of Boolean logic (using AND, OR, and NOT operators), incorporating a strategic combination of MeSH headings, such as (NSAIDs OR nonsteroidal anti-inflammatory agent) AND (renal insufficiency OR kidney insufficiency OR renal diseases). Furthermore, we retrieved the reference lists and citations included in the studies and searched the related literature.

#### 2.1.3 Eligibility criteria

##### 2.1.3.1 Types of studies

Observational studies, including cohort, case-control, and cross-sectional designs. The study only included English-language literature.

##### 2.1.3.2 Types of participants

Women exposed to NSAIDs during pregnancy.

##### 2.1.3.3 Observation results

The prevalence of kidney diseases in offspring.

##### 2.1.3.4 Exclusion criteria

(a) Reviews, case reports, animal studies, and conference abstracts.
(b) Studies with overlapping data (duplicates excluded).
(c) Studies without available results.

#### 2.1.4 Study selection

The titles and abstracts of all studies were independently reviewed by two reviewers (Feng and Cui), and when necessary, obtained the full text. The full text was reviewed for articles that met the following inclusion criteria: (1) cross-sectional studies, cohort, or case-control design; (2) studies on the association between the NSAIDs use of prenatal and kidney disease risk in children; and (3) studies with corresponding 95% confidence intervals (CIs) and odds ratios (ORs). The exclusion criteria were outlined as follows: (1) reviews, case reports, animal studies and conference abstracts; (2) research on data overlap (for example: excluded studies with similar data); and (3) studies with incomplete data (for example: the OR value cannot be calculated).

#### 2.1.5 Data extraction

Two independent reviewers (Feng and Cui) used standardized data collection forms to extract data from eligible studies. The extraction process was conducted systematically to ensure accuracy and consistency. Odds ratios (ORs) and 95% confidence intervals are used as effect estimators. Information extracted included the names of the authors, year of publication, details of the research design, country, study quality, sample size, adjusted confounding factors (if any), and adjusted estimates. Furthermore, the risk estimates for the association between AKI, congenital kidney abnormalities, or CKD were extracted separately in instances in which they were reported in the same paper. AKI was defined as functional or structural abnormalities of the kidneys, lasting no more than 3 months, caused by a variety of renal or extrarenal factors, including abnormal findings in renal injury markers from blood tests, urine tests, renal pathology examinations, and imaging studies. On the other hand, congenital kidney abnormalities were defined as structural abnormalities that occur during embryonic development and are present at birth. CKD was defined as an abnormality in the structure or function of the kidneys lasting for more than three months. Any discrepancies identified during the data extraction process were resolved by consensus with a third reviewer (Song).

#### 2.1.6 Quality assessment

Two researchers (Cui and Feng) assessed the potential risk of bias. Any differences were resolved through consensus with the involvement of third-party reviewers (Zhou). The methodological quality of cohort and case-control studies was evaluated using the Newcastle-Ottawa Scale (19). The maximum achievable score on this scale was 9. The quality of the studies was categorized into three levels: the high-quality score was 7–9 points, scores from 4 to 6 represented moderate quality, and scores from 0 to 3 indicated low quality. A cross-sectional study was conducted using the Assessment of Healthcare Quality and Research Institutions in the United States (20), with a total score of 11 points, also categorised into three grades: scores ranging from 8 to 11 indicated high quality, scores from 4 to 7 represented medium quality, and scores from 0 to 3 corresponded to low quality.

#### 2.1.7 Statistical analysis

The Review Manager (version 5.3; Cochrane, http://community.cochrane.org/tools/review-production-tools/revman-5/revman-5-download) and Stata (version 18.0; StataCorp) software programs were used for the data analysis. The relationship between maternal NSAIDs exposure and the risk of kidney disease in children was evaluated by ORs. The combined ORs of this study was calculated using the random effects model. We employed the *I*^2^ statistic (with statistical significance defined as *I*^2^ > 50%) and the *Q* statistic (with statistical significance defined as *P* < 0.10) to assess the heterogeneity across the studies (21). Subgroup analyses were stratified according to age, study design, study quality, and sample size. Furthermore, we performed a sensitivity analysis by sequentially excluding one study at a time to assess the robustness of our findings. The Begg's test was used to visually evaluate the possibility of publication bias (*P* < 0.05) (22). The impact of publication bias on meta-analysis was assessed using the Pruning and filling method. This approach examines how the magnitude of the corrected combined effect changes by estimating and “filling” studies that may be missing. It is used to identify and correct publication bias.

## 3 Results

### 3.1 Literature screening process

**Figure 1:**
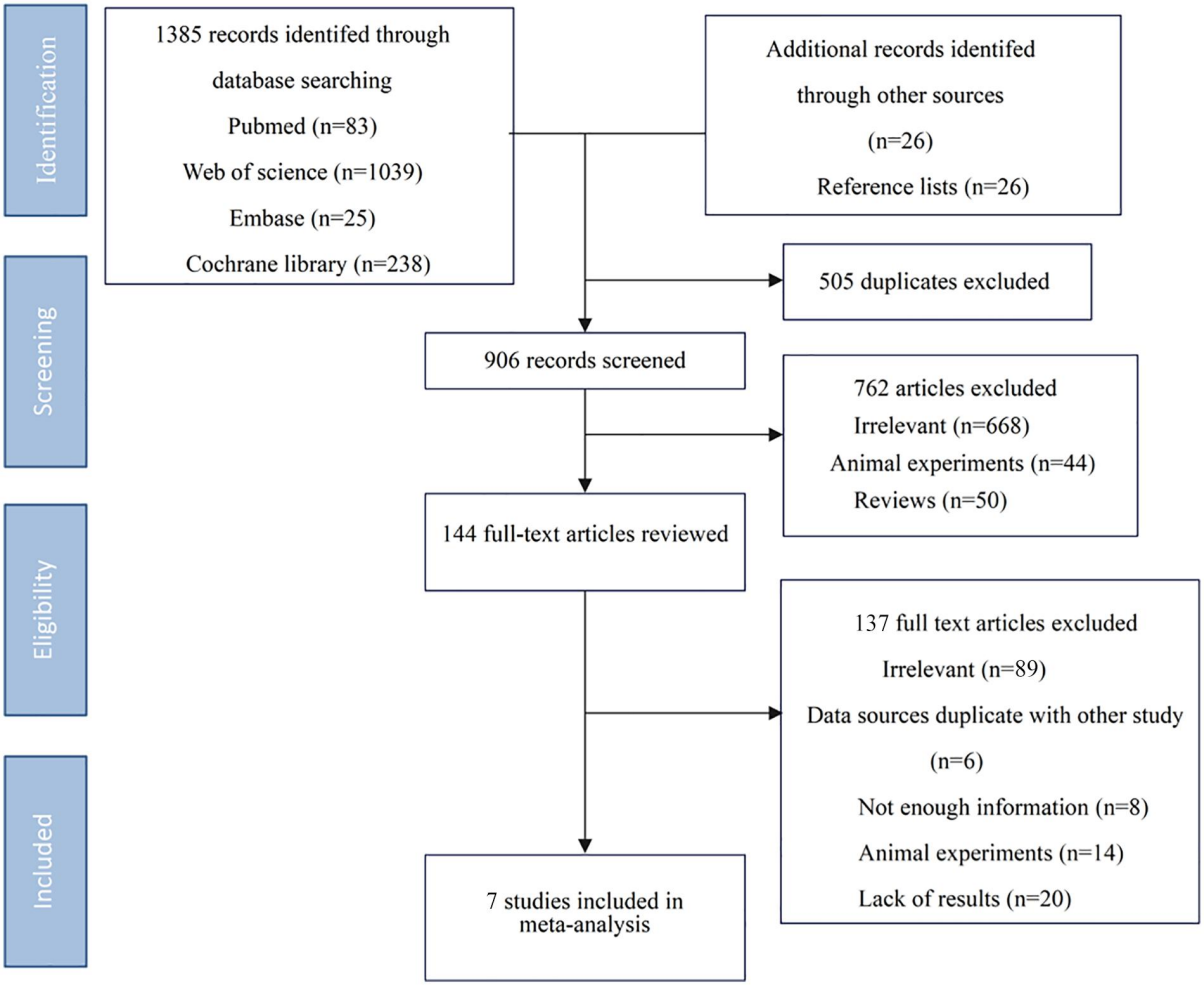
Flowchart of study inclusion and exclusion process.

In the initial literature search, 1,411 references were identified. After eliminating duplicates and filtering the abstracts and titles, 144 articles were reviewed. Of these, 137 articles did not meet the inclusion criteria, and the remaining seven articles were included in the analysis. The exclusion reasons are shown in Figure 1.

### 3.2 Characteristics of the included literature

Table 1 summarises the seven included studies, among which four were cohort studies (6, 11, 12, 16), and three were case-control studies (13–15).

**Table 1:** Characteristics of the included studies in the review.

Author, year	Location	Design	Definition of kidney disease	Participants	Kidney diseases cases	Age at pregnancy years (y)	Maternal race and ethnicity	Age range for children (days)	Period of gestational drug exposure	Effect estimates	Adjusted confounding factors	NOS
Cuzzolin et al. (2006) (13)	Italy	Case-control study	The absolute value of serum creatinine increased by ≥26.5 μmol/L (0.3 mg/dL) within 48 h	246	246	32.02 ± 3.62	White non-Hispanic	0–28	NA	NSAIDs: ORs = 7.38 (3.26–16.7)	Antibiotic, PROM	5
Choi et al. (2023) (11)	South Korea	Cohort study	The serum creatinine level rises to 1.5 times or more of the baseline value within 7 days	3,129,715	884	18–44	Asian	0–28	NA	COX–2: ORs = 1.19 (1.13–1.24)	Age, multiple gestation, diabetes, gastrointestinal diseases, renal disease, respiratory infection, antibiotics, steroids, fever	8
Charlton et al. (2018) (12)	US	Cohort study	The serum creatinine level rises to 1.5 times or more of the baseline value within 7 days	2,152	202	NA	Hispanic	0–28	NA	NSAIDs: ORs = 2.58 (1.22–5.45)	Multiple gestation, antibiotics, steroids for fetal maturation, hypertensive disease during pregnancy, mode of delivery	7
Tain et al. (2024) (6)	Tai wan	Cohort study	The presence of markers of kidney damage, persisting for more than 3 months	1,025,255	NA	25–45	Asian	NA	Second-trimester: Diclofenac: ORs = 1.17 (1.09–1.26)	ORs = 1.10 (1.05–1.15)	Age, antimicrobial agents, gestational diabetes, gestational hypertension, preeclampsia	9
										Diclofenac: ORs = 1.27 (1.13–1.42)		
									Third-trimester: Ibuprofen: ORs = 1.10 (1.09–1.11)			
										Ketorrole acid: ORs = 1.28 (1.01–1.62)		
										Mefenac: ORs = 1.29 (1.15–1.46)		
										Ibuprofen: ORs = 1.34 (1.07–1.68)		
Dathe et al. (2023) (16)	German	Cohort study	Unilateral kidney hypoplasia	1,154	20	15–49	White non-Hispanic	0–28	Second-trimester: NSAIDs: ORs = 4.23 (0.90–19.88)	NSAIDs: ORs = 1.00 (0.10–16.5)	Educational level, smoking, alcohol, previous deliveries	6
									Third-trimester: NSAIDs: ORs = 1.21 (0.66–2.22)			
Sanderson et al. (2024) (15)	US	Case-control study	The absolute value of serum creatinine increased by ≥26.5 μmol/L (0.3 mg/dL) within 48 h	923	386	28.9 ± 6.2	Hispanic	0–28	NA	NSAIDs: ORs = 3.16 (1.02–9.81)	Mode of delivery, maternal education status, antenatal steroids, alcohol, tobacco, gestational diabetes, preeclampsia, gestational hypertension	8
Cataldi et al. (2013) (14)	Italy	Case-control study	The absolute value of serum creatinine increased by ≥26.5 μmol/L (0.3 mg/dL) within 48 h	172	71	31.03 ± 5.64	White non-Hispanic	0–28	NA	ORs = 2.30 (1.04–5.07)	Renal disease during pregnancy, smoking, steroids, alcohol, preeclampsia, fever, caesarean section, twins, antibiotics	5

NOS, Newcastle-Ottawa scale; NA, not applicable; OR, odds ratio; PROM, premature rupture of membranes; US, United States; AKI, acute kidney injury; CKD, chronic kidney disease.

No appropriate cross-sectional studies were identified. Among the included studies, four were considered high quality (6, 11, 12, 15) and three were considered moderate quality (13, 14, 16).

### 3.3 NSAIDs use during pregnancy and kidney disease meta-analysis

**Figure 2:**
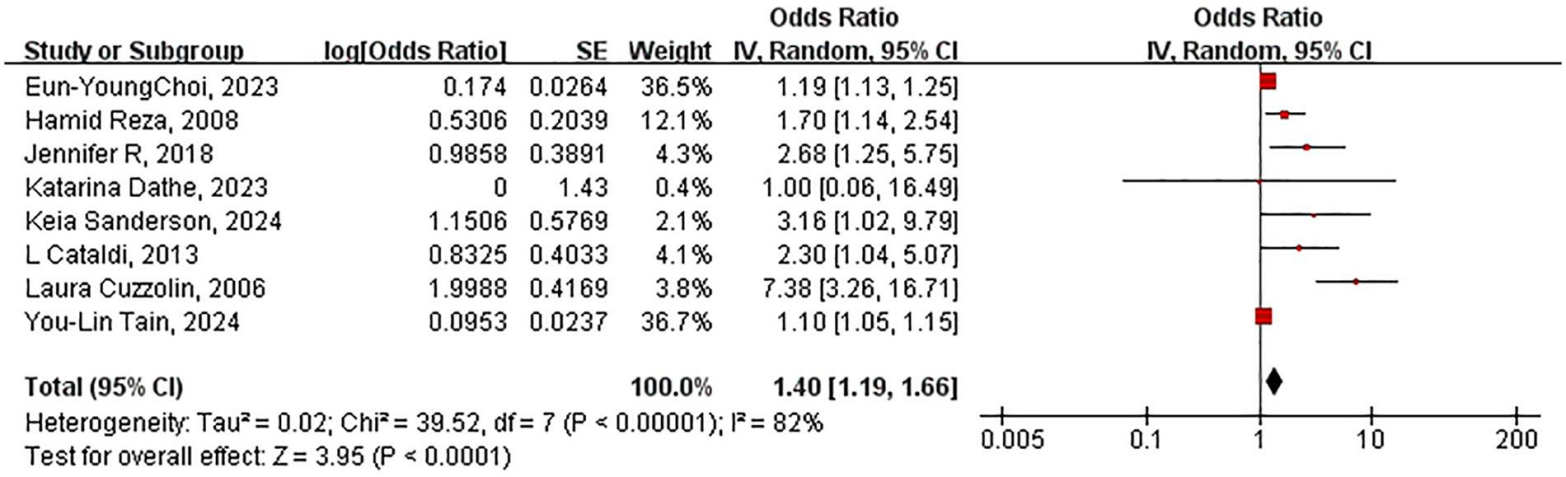
Forest plot of the association between NSAIDs exposure during pregnancy and kidney disease in offspring. OR, odds ratio; CI, confidence interval. The total sample size was 4,159,617 with NSAIDs exposure during the entire pregnancy.

Overall, 4,159,617 participants were included in seven studies (6, 11–16), and meta-analysis revealed an association between NSAIDs exposure of prenatal and kidney disease risk in children (OR: 1.36 95% CI: 1.14–1.62) (Table 2). Substantial heterogeneity was observed among these studies (*I*^2^ = 83%; *P* < 0.00001) (Figure 2). According to the sensitivity analysis, after excluding any one study, the overall a ggregated results did not significantly change (Table 3).

**Table 2:** Meta-analysis results of the association between prenatal NSAIDs exposure and childhood kidney disease, test of publication bias and trim and fill analysis.

Category	No. of studies	Effect size	Heterogeneity		Publication bias		Trim-and-fill method	
		OR (95% CI)	*I*^2^, %	*P*	Begg's test (*z*, *P*)	Egger's test (*t*, *P*)	No. of potential missing studies	Filled estimates
Use NSAIDs during pregnancy	7	1.36 (1.14–1.62)	83	<0.0001	0.15, 1.00	2.13, 0.086	3	1.19 (1.11–1.47)
Adjusted results	3	2.40 (1.84–3.13)	86	0.0007	–	–	–	–
Unadjusted results	4	1.11 (1.06–1.16)	88	<0.0001	–	–	–	–
Use NSAIDs in the second-trimester	2	1.17 (1.09–1.26)	0	0.1	–	–	–	–
Use NSAIDs in the third-trimester	2	1.10 (1.09–1.11)	0	0.75	–	–	–	–

NSAIDs, nonsteroidal anti-inflammatory drugs; OR, odds ratio; Second-trimester refers to 13–26 weeks; Third-trimester refers to 27 weeks or more.

**Table 3:** Sensitivity analysis of the association between prenatal NSAIDs exposure and childhood kidney disease.

Study excluded	Effect size	Heterogeneity	
	OR (95% CI)	*I*^2^, %	*P*
Unadjusted OR of maternal NSAIDs and kidney diseases			
Choi et al. 2023 (11)	2.47 (1.17–5.25)	85	<0.00001
Charlton et al. 2018 (12)	1.30 (1.09–1.54)	84	<0.00001
Dathe et al. 2023 (16)	1.36 (1.14–1.63)	86	<0.00001
Sanderson et al. 2024 (15)	1.32 (1.11–1.57)	85	<0.00001
Cataldi et al. 2013 (14)	1.32 (1.10–1.57)	85	<0.00001
Cuzzolin et al. 2006 (13)	1.19 (1.06–1.43)	68	0.007
Tain et al. 2024 (6)	2.51 (1.24–5.11)	83	<0.00001
Adjusted OR of maternal NSAIDs and kidney diseases			
Choi et al. 2023 (11)	4.35 (2.88–6.57)	0	<0.00001
Sanderson et al. 2024 (15)	2.31 (1.75–3.06)	93	<0.00001
Charlton et al. 2018 (12)	1.76 (1.28–2.42)	61	0.0005

NSAIDs, nonsteroidal antiinflammatory drugs; OR, odds ratio.

**Figure 3:**
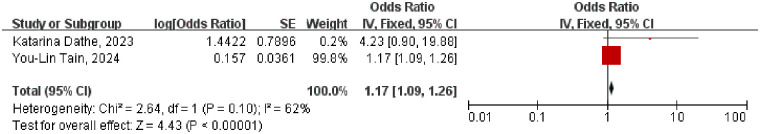
Forest plot of the association between NSAIDs exposure during second-trimester and kidney disease in offspring. OR, odds ratio; CI, confidence interval. The total sample size was 55,126 with NSAIDs exposure during second-trimester.

For second-trimester NSAIDs exposure, a meta-analysis of two studies (55,126 children) (6, 16) estimated an association between second-trimester NSAIDs exposure and kidney disease in children (OR: 1.17, 95% CI: 1.09–1.26). No significant heterogeneity was observed between the studies (*I*^2^ = 62%, *P* = 0.1) (Figure 3).

**Figure 4:**
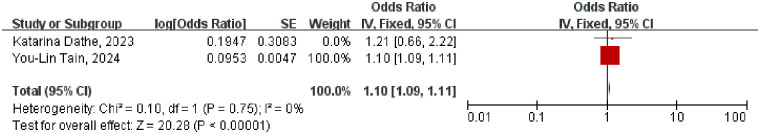
Forest plot of the association between NSAIDs exposure during third-trimester and kidney disease in offspring. OR, odds ratio; CI, confidence interval. The total sample size was 55,126 with NSAIDs exposure during third-trimester.

Regarding the third-trimester, a meta-analysis of two studies (55,126 children) (6, 16) estimated an association between third-trimester NSAIDs exposure and kidney disease in children (OR: 1.10, 95% CI: 1.09–1.11). No significant heterogeneity was observed (*I*^2^ = 0%, *P* = 0.75) (Figure 4).

Table 4 shows the detailed outcomes of the subgroup analysis. The pooled OR for the four cohort studies (6, 11, 12, 16) was 1.11 (95% CI: 1.08–1.15). The pooled OR of the three case-controls studies (13–15) was 2.85 (95% CI: 1.21–4.48). Four high-quality studies (6, 11, 12, 15) had a combined OR of 1.11 (95% CI: 1.08–1.15), while three moderate-quality studies (13, 14, 16) had a combined OR of 2.63 (95% CI: 1.75–4.50). Three studies (11, 12, 15) that adjusted for confounding factors (for example: age, multiple pregnancies, respiratory tract infections and fever, etc.) reported a combined OR of 2.40 (95% CI: 1.84–3.13), while four studies (6, 13, 14, 16) that did not account for confounding factors had a combined OR of 1.12 (95% CI: 1.07–1.17). Five studies (6, 11–14) adjusted for antibiotics use in pregnancy had a combined OR of 3.51 (95% CI: 3.47–3.54), while two studies (15, 16) with unadjusted antibiotics use had a combined OR of 2.68 (95% CI: 1.2–6.55). Gestational steroid use was adjusted for in four (11, 12, 14, 16) with a combined OR of 4.01 (95% CI: 4.05–4.15), while three studies (6, 13, 15) that were unadjusted and had a combined OR of 1.05 (95% CI: 1.0–1.1). Two studies (11, 12) adjusted for fevers in pregnancy had a combined OR of 3.36 (95% CI: 3.26–3.46), while five studies (6, 13–16) that were unadjusted resulted in an OR of 1.05 (95% CI: 1.0–1.10). Three studies (6, 14, 15) that adjusted for preeclampsia had a combined OR of 2.90 (95% CI: 2.62–3.19), while four studies (11–13, 16) were unadjusted and had the same OR of 1.19 (95% CI: 1.14–1.25).

**Table 4:** Subgroup analysis of the association between prenatal NSAIDs exposure and childhood kidney disease.

Variables	Adjusted results					Unadjusted results				
	No. of studies	Effect size OR (95%CI)	Heterogeneity		*P* for the subgroup	No. of studies	Effect size OR (95%CI)	Heterogeneity		*P* for the subgroup
			*I*^2^, %	*P*				*I*^2^, %	*P*	
Study design										
Cohort	2	1.19 (1.14–1.25)	40	0.198	0.036	2	1.10 (1.05–1.15)	0	0.981	0.358
Case-control	2	2.72 (1.79–4.65)	50	0.156		1	3.16 (1.02–9.81)	–	–	
Study quality										
Moderate	–	–	–	–	–	3	1.63 (1.35–2.50)	8	0.337	0.042
High	3	1.19 (1.14–1.25)	17.7	0.297		1	1.10 (1.05–1.15)	–	–	
Sample size										
<10,000	2	2.69 (1.78–4.59)	0	0.816	0.037	3	1.71 (1.08–2.17)	0	0.425	0.789
≥10,000	1	1.19 (1.13–1.24)	–	–		1	1.10 (1.05–1.15)	–	–	
Age of study participants										
<25years	–	1.29 (1.28–1.31)	99.3	<0.001	<0.00001	–	–	–	–	–
25–35years	–	1.02 (1.02–1.03）	67.5	0.08		–	–	–	–	
Kidney disease definition										
Increased creatinine	5	2.61 (1.29–5.27）	87	<0.001	<0.00001	–	–	–	–	–
Congenital kidney abnormality	1	1.00 (0.10–16.5）	–	–		–	–	–	–	
Chronic kidney disease	1	1.10 (1.05–1.15）	–	–		–	–	–	–	
Gestational exposure to different types of NSAIDs										
Ibuprofen	–	–	–	–	–	4	1.47 (1.31–1.63)	13.7	0.324	0.011
Diclofenac	–	–	–	–		2	1.09 (0.97–1.21)	94.3	<0.001	
Adjusted for maternal gestational diabetes										
Yes	3	1.07 (1.04–1.09)	99.5	<0.001	<0.00001	–	–	–	–	–
No	4	2.61 (1.02–4.01)	0	0.537		–	–	–	–	
Adjusted for Preeclampsia										
Yes	3	2.90 (2.62–3.19)	98.7	<0.001	<0.00001	–	–	–	–	–
No	4	1.19 (1.14–1.25)	39	0.178		–	–	–	–	
Adjusted for multiple gestation										
Yes	3	1.12 (1.07–1.16)	92.9	<0.001	<0.00001			–	–	–
No	4	1.05 (1.00–1.10)	30.1	0.231				–	–	
Adjusted for maternal fever										
Yes	2	3.36 (3.26–3.46)	0	0.452	<0.00001			–	–	–
No	5	1.05 (1.00–1.10)	36.5	0.178				–	–	
Adjusted for maternal renal disease										
Yes	2	1.89 (1.72–2.06)	86.4	0.007	0.001			–	–	–
No	5	1.05 (1.00–1.10)	36.5	0.178				–	–	
Adjusted for maternal steroid exposure										
Yes	4	4.10 (4.05–4.15)	99.5	<0.001	<0.00001			–	–	–
No	3	1.05 (1.00–1.10)	41.3	0.182				–	–	
Adjusted for maternal antibiotics exposure										
Yes	5	3.51 (3.47–3.54)	99.9	<0.001	<0.00001			–	–	–
No	2	2.68 (1.20–6.55)	0	0.649				–	–	
Adjusted for maternal age										
Yes	3	1.08 (1.08–1.09)	93.3	<0.001	<0.00001			–	–	–
No	4	2.61 (1.20–4.01)	0	0.537				–	–	

Unadjusted effect sizes indicate a crude exposure-outcome association that may be confounded by factors such as age or comorbidities. However, adjusted effect sizes, statistically account for these known confounders and thus reflect a more valid approximation of the true relationship. Consequently, we prioritized adjusted estimates in our meta-analysis as they provide more reliable evidence with lower potential for bias. In our subgroup analyses, some of the adjusted estimates appeared larger, which may be due to factors such as model instability, inconsistent covariate sets, over-adjustment, or collider bias.

### 3.4 Sensitivity analysis

We conducted a sensitivity analysis by sequentially omitting each study. The results consistently showed stable outcome estimates, demonstrating the robustness of the pooled ORs. These findings confirmed the stability of the meta-analysis and the association between NSAIDs exposure of prenatal and kidney diseases risk in children.

### 3.5 Publication bias

**Figure 5:**
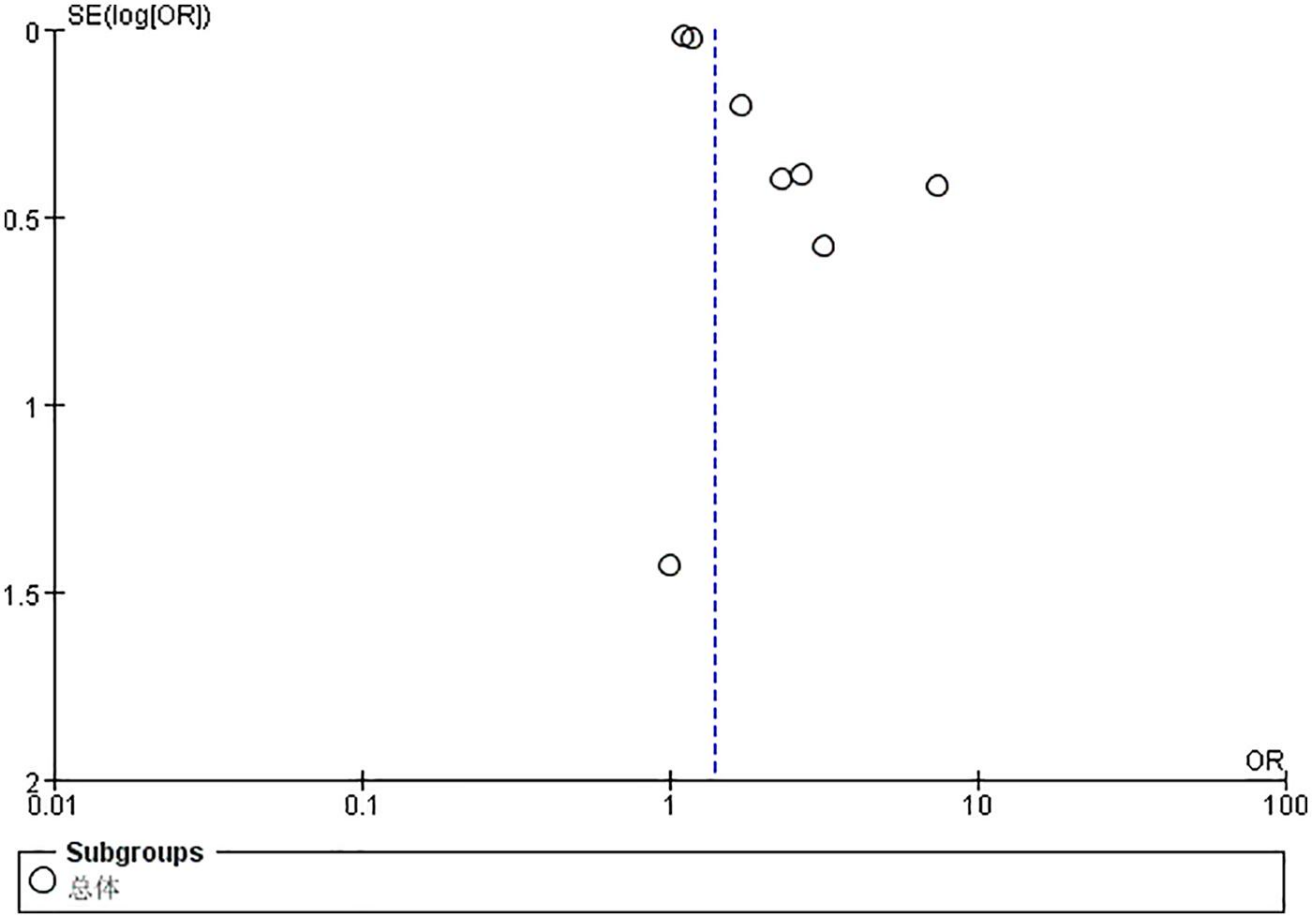
Funnel plot of the association between NSAIDs exposure during pregnancy and kidney disease in offspring. OR, odds ratio; SE, standard error. The total sample size was 4,159,617 with NSAIDs exposure during the entire pregnancy.

Table 2 presents the results of the publication bias and trim-and-fill analyses. The visual inspection of the funnel plot showed the presence of potential publication bias. (Figure 5). Pruning and filling methods were used to estimate the number of potentially missing studies that may have driven the observed asymmetry. The trim-and fill analysis indicated that three studies on NSAIDs exposure of prenatal were missing and contributed to asymmetry of the funnel plot. However, the analysis of missing value estimation did not produce different results [total NSAIDs exposure: OR 1.36 (95% CI: 1.14–1.62); after trimming and filling: 1.19 (95% CI: 1.11–1.47)]. Table 5 shows the quality assessment of studies. The assessment of Immortal Time Bias for the eight included studies revealed that the majority (*n* = 7) were at low risk (Table 6). These studies appropriately handled the timing of NSAID exposure, predominantly using time-dependent Cox models. The risk for the remaining study was unclear due to insufficient description of the analytical methods. Additionally, a meta analysis to these seven low-risk articles showed that the results remained statistically significant (OR = 1.35; 95% CI: 1.14–1.61).

**Table 5:** Quality assessment of studies included according to the NOS.

Study, year	Selection ① Is the case definition adequate? ② Representativeness of the cases ③ Selection of Controls ④ Definition of Controls	Comparability ① study controls for the most important factor (specify) ②study controls for any additional factor (specify)	Exposure ① Ascertainment of exposure ② Same method of ascertainment for cases and controls ③ Non-Response rate	Total	Overall quality
Cuzzolin et al., 2006 (13)	①, ②	①	①, ②	5	Moderate
Choi et al., 2023 (11)	①, ②, ③, ④	①, ②	①, ③	8	High
Charlton et al., 2018 (12)	①, ②, ③	①, ②	①, ③	7	High
Tain et al., 2024 (6)	①, ②, ③, ④	①, ②	①, ②, ③	9	High
Dathe et al., 2023 (16)	①, ②, ③	①	①, ③	6	Moderate
Sanderson et al., 2024 (15)	②, ③, ④	①, ②	①, ②, ③	8	High
Cataldi et al., 2013 (14)	③, ④	①	①, ②	5	Moderate

Key: the high-quality score was 7–9 points, scores from 4 to 6 represented moderate quality, and scores from 0 to 3 indicated low quality. NOS, Newcastle-Ottawa scale.

**Table 6:** Assessment of immortal time bias across included literature.

Study	Immortal Time Bias	Appropriate methods	Risk assessment
Cuzzolin et al. (2006) (13)	Yes	Time-dependent Cox analysis	Low risk
Choi et al. (2023) (11)	Yes	Time-dependent Cox analysis	Low risk
Charlton et al. (2018) (12)	Yes	Time-dependent Cox analysis	Low risk
Tain et al. (2024) (6)	Yes	Time-dependent Cox analysis	Low risk
Dathe et al. (2023) (16)	Yes	Time-dependent Cox analysis	Low risk
Sanderson et al. (2024) (15)	Yes	Time-dependent Cox analysis	Low risk
Cataldi et al. (2013) (14)	Yes	Time-dependent Cox analysis	Low risk

## 4 Discussion

### 4.1 Summary of main findings

In this meta-analysis, we systematically assessed the association between NSAIDs exposure of prenatal and kidney diseases risk in children. The results revealed an association between the NSAIDs use of prenatal and kidney disease in children. To our knowledge, no previous systematic review has explored the association between maternal exposure to NSAIDs and the risk of kidney disease in children.

### 4.2 Underlying mechanisms linking prenatal NSAID exposure and childhood kidney disease

These findings are biologically plausible for a number of reasons. Firstly, NSAIDs can inhibit the synthesis of prostaglandins (PG). Most PG synthases and receptors are expressed in the early stage of human fetal kidneys (23, 24). Secondly, cyclooxygenase-2 (COX-2) enzyme is widely and significantly expressed in the developing fetal kidneys, first appearing in the second trimester of pregnancy and gradually increasing in the third trimester (5, 25). COX-2 is involved in the proliferation and differentiation of renal tubular epithelial cells and plays an important role in renal tubular function (24, 26).

Despite the significant findings, potential confounding factors should still be considered. For example, the original research results may have been influenced by factors, such as the use of antibiotics, use of steroids, fever during pregnancy and maternal preeclampsia. In our subgroup analysis, the results showed that there was an association between the antibiotics used during pregnancy and kidney disease in children. These results remained significant after adjusting for antibiotics use during pregnancy. Our subgroup analysis showed that there was no marked difference in the risk of kidney disease among the children, regardless of steroids use, maternal fever, or maternal preeclampsia during pregnancy. Some subgroups only had two studies, these pooled results should be interpreted with caution and are best suited for hypothesis generation rather than for establishing causal relationships.

Several factors suggest a positive correlation between NSAIDs use of prenatal and kidney disease in children. First, our research indicates that there was an association between NSAIDs exposure in the second and third trimesters of pregnancy and the risk of kidney disease in children. Second, there was an association between the use of different drugs, such as ibuprofen and diclofenac, during pregnancy and the risk of kidney disease in children. Finally, after adjusting for possible confounding factors, there was also an association between the NSAIDs use during pregnancy and kidney disease risk in children.

Our analysis revealed significant heterogeneity (*I*^2^ > 50%). Therefore, we conducted a series of subgroup analyses to explore potential sources. We stratified data by research design (cohort vs. case-control), sample size, specific drug (Ibuprofen vs. Diclofenac), study quality (moderate vs. high), definition of kidney disease, and confounding factors. We found that heterogeneity was reduced within the case-control subgroup, likely attributable to the inherent methodological differences between study designs. Similarly, heterogeneity was lower in studies with larger sample sizes, suggesting that the robust design of larger studies enables more comprehensive control of confounding variables. Subgroup analysis based on congenital kidney abnormality also showed reduced heterogeneity, which may be explained by distinct pathogenic mechanisms and outcome indicators. Furthermore, heterogeneity was low in subgroups defined by Ibuprofen use, high study quality, and maternal fever. The notably high ORs observed in the adjusted subgroups are likely due to over-adjustment or the instability associated with rare events. Future studies adjusting for these confounding factors are necessary to further explore these associations. For some subgroup analyses, the number of studies was small (e.g., antibiotic use), and higher-quality studies are needed for subgroup analyses in the future.

We conducted meta-regression analyses to examine the potential influence of several factors on the results, including study type, study quality, specific drug, exposure window, and the definition of kidney disease. The meta-regression analysis identified the type of drug, exposure window, and definition of kidney disease as major sources of heterogeneity (Table 7).

**Table 7:** Meta-regression analysis of prenatal NSAIDs exposure and childhood kidney disease.

Variable	Coef	*P*	95% CI	
Research type	0.430	0.211	−0.294	1.153
Research quality	0.427	0.192	−0.301	1.156
Drug type	0.181	0.004	0.090	0.273
Exposure time window	0.118	0.038	0.012	0.224
Diagnosis of kidney diseases	0.232	0.035	0.352	0.816

This paper also has notable advantages. This meta-analysis assessed the association between NSAIDs exposure of prenatal and kidney disease in children, adhering to the recommended guidelines to ensure methodological rigour. Our research included 4,160,723 participants, with extensive global and trans-national representation. Moreover, the results remained consistent throughout the sensitivity analysis, indicating robustness and reliability of the findings.

However, this study has some limitations. First, the use of NSAIDs may be subject to recall bias, confounding by indication is likely and difficult to fully control in registry-based and questionnaire-based studies. Second, significant heterogeneity indicated methodological differences between the included studies. Third, residual confounding factors, which were not fully adjusted for, may introduced bias into the results. Fourth, the small number of studies and lack of randomized experiments may affect the robustness of the study, lack of unified kidney disease definition could have contributed to the heterogeneity of study, observational nature of included studies may be difficult to fully identify and control all possible confounding factors. Finally, the included studies did not mention immortal time bias. Its potential presence may affect the estimated effect of the exposure.

In this meta-analysis, only one study specifically examined the association between the risk of maternal prenatal exposure to NSAIDs and childhood chronic kidney disease (6), which limited our ability to analyse this subgroup. Only two studies (6, 16) focused on the relationship between mothers’ exposure to NSAIDs in the second and third trimesters of prenatal and kidney diseases in their children. More high-quality studies are necessary to study the relationship between mothers’ exposure to NSAIDs in the second and third trimesters of prenatal and kidney diseases in children. Four studies have focused on the relationship between maternal exposure to different types of NSAIDs of prenatal and kidney disease in children (6, 13, 14, 16). Only two studies have focused on the effects of the duration of NSAIDs use of prenatal (<5, 5–10, and >10 days) on the kidneys of children (11, 16). At present, there are few studies on subgroups of these factors. More larger prospective studies are needed in the future to focus on the relationship between the exposure period, the usage duration of NSAIDs and the type of NSAIDs used, and unified kidney outcomes in children. Meanwhile, research should also focus on the exposure period, types, and duration of NSAIDs used during pregnancy. Finally, the results of this meta-analysis underscore the critical importance of healthcare practitioners exercising heightened caution and clinical judgment when managing medication use during pregnancy. Higher-quality prospective studies are needed, which are dedicated to conducting detailed subgroup analyses and strictly controlling confounding factors to determine the relationship between NSAIDs use of prenatal and kidney disease in children.

## 5 Conclusion

This study suggests an association between prenatal NSAIDs exposure and kidney diseases in children but do not solve the causality issues concerning potential confounding by other risk factors. These associations need to be interpreted cautiously. More high-quality studies are needed to establish whether the association with NSAIDs is causal.

## Data Availability

The original contributions presented in the study are included in the article/Supplementary Material, further inquiries can be directed to the corresponding author.

## References

[1] Ingelfinger JR Kalantar-Zadeh K Schaefer F, World Kidney Day Steering Committee. Averting the legacy of kidney disease—focus on childhood. Saudi J Kidney Dis Transpl. (2016) 27(2):219–26. 10.4103/1319-2442.178201 26997373

[2] Harambat J Madden I. What is the true burden of chronic kidney disease in children worldwide? Pediatr Nephrol. (2023) 38(5):1389–93. 10.1007/s00467-022-05816-7 36409363

[3] Parikh RV Tan TC Salyer AS Auron A Kim PS Ku E Community-based epidemiology of hospitalized acute kidney injury. Pediatrics. (2020) 146(3):e20192821. 10.1542/peds.2019-2821 32784225 PMC7461200

[4] Eckardt K-U Coresh J Devuyst O Johnson RJ Köttgen A Levey AS Evolving importance of kidney disease: from subspecialty to global health burden. Lancet. (2013) 382:158–69. 10.1016/S0140-6736(13)60439-0 23727165

[5] Jacobson MH Wu Y Liu M Kannan K Li AJ Robinson M Organophosphate pesticides and progression of chronic kidney disease among children: a prospective cohort study. Environ Int. (2021) 155:106597. 10.1016/j.envint.2021.106597 33951537 PMC8292180

[6] Tain YL Li LC Kuo HC Chen CJ Hsu CN. Gestational exposure to nonsteroidal anti-inflammatory drugs and risk of chronic kidney disease in childhood. JAMA Pediatr. (2024) 179(2):171–8. 10.1001/jamapediatrics.2024.4409 PMC11791701 39714827

[7] Powers EA Tewell R Bayard M. Over-the-counter medications in pregnancy. Am Fam Physician. (2023) 108(4):360–9. 37843943

[8] Vroom F van den Berg PB de Jong-van den Berg LT. Prescribing of NSAIDs and ASA during pregnancy: do we need to be more careful? Br J Clin Pharmacol. (2008) 65(2):275–6. 10.1111/j.1365-2125.2007.02994.x 18251762 PMC2291219

[9] Lupattelli A Spigset O Twigg MJ Zagorodnikova K Mårdby AC Moretti ME Medication use in pregnancy: a cross-sectional, multinational web-based study. BMJ Open. (2014) 4(2):e004365. 10.1136/bmjopen-2013-004365 24534260 PMC3927801

[10] Oma A Omar H Mohamad A. Non-steroidal anti-inflammatory drugs consumption and awareness about risks during pregnancy in AL-bayda city, Libya. Alq J Med App Sci. (2025) 8(1):119–28. 10.54361/ajmas.258119

[11] Choi E-Y Jeong HE Noh Y Choi A Yon DK Han JY Neonatal and maternal adverse outcomes and exposure to nonsteroidal anti-inflammatory drugs during early pregnancy in South Korea: a nationwide cohort study. PLoS Med. (2023) 20(2):e1004183. 10.1371/journal.pmed.1004183 36848338 PMC9970080

[12] Charlton JR Boohaker L Askenazi D Brophy PD Fuloria M Gien J Late onset neonatal acute kidney injury: results from the AWAKEN study. Pediatr Res. (2018) 85(3):339–48. 10.1038/s41390-018-0255-x 30546043 PMC6438709

[13] Cuzzolin L Fanos V Pinna B di Marzio M Perin M Tramontozzi P Postnatal renal function in preterm newborns: a role of diseases, drugs and therapeutic interventions. Pediatr Nephrol. (2006) 21:931–8. 10.1007/s00467-006-0118-2 16773403

[14] Cataldi L Leone R Moretti U De Mitri B Fanos V Ruggeri L Potential risk factors for the development of acute renal failure in preterm newborn infants: a case-control study. Arch Dis Child Fetal Neonatal Ed. (2005) 90(6):F514–9. 10.1136/adc.2004.060434 16244211 PMC1721962

[15] Sanderson K Griffin R Anderson N South AM Swanson JR Zappitelli M Perinatal risk factors associated with acute kidney injury severity and duration among infants born extremely preterm. Pediatr Res. (2024) 96(3):740–9. 10.1038/s41390-024-03102-w 38438550 PMC11371939

[16] Dathe K Frank J Padberg S Hultzsch S Beck E Schaefer C. Fetal adverse effects following NSAID or metamizole exposure in the 2nd and 3rd trimester: an evaluation of the German embryotox cohort. BMC Pregnancy Childbirth. (2023) 22(1):666. 10.1186/s12884-022-04986-4 PMC9413886 36028798

[17] Sutherland MR Yoder BA McCurnin D Seidner S Gubhaju L Clyman RI Effects of ibuprofen treatment on the developing preterm baboon kidney. Am J Physiol Renal Physiol. (2012) 302:F1286–92. 10.1152/ajprenal.00216.2011 22357916 PMC3362063

[18] Stroup DF Berlin JA Morton SC Olkin I Williamson GD Rennie D Metaanalysis of observational studies in epidemiology: a proposal for reporting. Metaanalysis of observational studies in epidemiology (MOOSE) group. J Am Med Assoc. (2000) 283:2008–12. 10.1001/jama.283.15.2008 10789670

[19] Stang A. Critical evaluation of the Newcastle-Ottawa scale for the assessment of the quality of nonrandomized studies in meta-analyses. Eur J Epidemiol. (2010) 25(9):603–5. 10.1007/s10654-010-9491-z 20652370

[20] Rostom A Dube C Cranney A. Celiac Disease. Appendix D. Quality Assessment Forms. Evidence Reports/Technology Assessments, No 104. Rockville (MD): Agency for Healthcare Research and Quality (US) (2004).

[21] Higgins JP Thompson SG Deeks JJ Altman DG. Measuring inconsistency in meta-analyses. Br Med J. (2003) 327(7414):557–60. 10.1136/bmj.327.7414.557 12958120 PMC192859

[22] Duval S Tweedie R. Trim and fill: a simple funnel-plot-based method of testing and adjusting for publication bias in meta-analysis. Biometrics. (2000) 56:455–63. 10.1111/j.0006-341x.2000.00455.x 10877304

[23] D’Ambrosio V Vena F Scopelliti A D’Aniello D Savastano G Brunelli R Use of non-steroidal anti-inflammatory drugs in pregnancy and oligohydramnios: a review. J Matern Fetal Neonatal Med. (2023) 36(2):2253956. 10.1080/14767058.2023.2253956 38092425

[24] Ding H Zhang L Yang Q Zhang X Li X. Epigenetics in kidney diseases. Adv Clin Chem. (2021) 104:233–97. 10.1016/bs.acc.2020.09.005 34462056 PMC9322755

[25] Jia X Zhu L Zhu Q Zhang J. The role of mitochondrial dysfunction in kidney injury and disease. Autoimmun Rev. (2024) 23(6):103576. 10.1016/j.autrev.2024.103576 38909720

[26] Smith FG Wade AW Lewis ML Qi W. Cyclooxygenase (COX) inhibitors and the newborn kidney. Pharmaceuticals. (2012) 5(11):1160–76. 10.3390/ph5111160 24281306 PMC3816666

